# ﻿A new species of Zoraptera, *Zorotypuskomatsui* sp. nov. from Cameroon and a redescription of *Zorotypusvinsoni* Paulian, 1951 (Polyneoptera, Zoraptera)

**DOI:** 10.3897/zookeys.1178.108276

**Published:** 2023-09-01

**Authors:** Yoko Matsumura, Munetoshi Maruyama, Nelson N. Ntonifor, Rolf G. Beutel

**Affiliations:** 1 Systematic Entomology, Department of Ecology and Systematics, Graduate School of Agriculture, Hokkaido University, Sapporo, Japan Hokkaido University Sapporo Japan; 2 The Kyushu University Museum, Hakozaki 6-10-1, Fukuoka, 812–8581, Japan The Kyushu University Museum Fukuoka Japan; 3 Department of Agronomic and Applied Molecular Sciences, Faculty of Agriculture and Veterinary Medicine, University of Buea, P.O. Box 63 Buea, Cameroon University of Buea Buea Cameroon; 4 Entomology Group, Institut für Zoologie und Evolutionsforschung, Friedrich-Schiller-Universität Jena, Jena, Germany Friedrich-Schiller-Universität Jena Jena Germany

**Keywords:** Afrotropical region, ground lice, Madagascar, new distribution record, taxonomy

## Abstract

A new species of the order Zoraptera, *Zorotypuskomatsui* Matsumura, Maruyama, Ntonifor & Beutel, **sp. nov.**, is described from Cameroon. The female and male morphology of another species, *Z.vinsoni*, is re-described, and its new distribution in Madagascar is recorded. A particular focus is on the male postabdominal morphology. This is apparently a crucial body region in the very small order with an extreme variation of the genital apparatus but otherwise a very uniform morphology. The male of the newly described species shares rudimentary male genitalia and well-developed postabdominal projections with the distantly related *Spermozorosimpolitus*, apparently a result of parallel evolution. Whether males of *Z.komatsui* also perform external sperm transfer like *S.impolitus* remains to be shown. The collecting of the material used for this study suggests that the present knowledge of zorapteran species diversity of the Afrotropical region is very fragmentary.

## ﻿Introduction

Together with Dermaptera (earwigs), the small order Zoraptera (ground lice) likely forms the sister clade of the entire remaining Polyneoptera (Misof et al. 2014; Wipfler et al. 2019). The crown species originated ca 270 Ma (Matsumura et al. 2020), and the origin of the order goes back to the Paleozoic (Evangelista et al. 2019; Matsumura et al. 2020). Ground lice are characterized by a morphological and behavioral megadiversity in reproductive features, including even sperm ultra­structure (Hünefeld 2007; Dallai et al. 2012a, 2012b, 2014a, 2014c; Matsumura et al. 2014: genitalic structures; Choe 1994, 1995, 1997; Dallai et al. 2013: mating behavior; Dallai et al. 2011, 2012a, 2014a, 2014b, 2014c: sperm ultrastructure). This is in stark contrast to the far-reaching uniformity of their general morphology.

The order presently comprises only 46 extant species (Kočárek and Horká 2023a) and is thus the third smallest after Grylloblattodea and Mantophasmatodea (Beutel et al. 2014; Mashimo et al. 2014; Wipfler et al. 2014; Choe 2018; Eberhard et al. 2018). Their diversification might have been inhibited due to a combination of miniaturization, structural simplification, and morphological specialization (Matsumura et al. 2022). However, as pointed out by Mashimo et al. (2014, 2018) and Choe (2018), the true species diversity has not been unveiled yet and more and more species have been discovered recently (e.g., Kočárek and Horká 2022, 2023a, 2023b; Kočárek and Hu 2023). So far only six out of 46 extant species are known from the Afrotropical region (Mashimo et al. 2018). Considering that 20 species are known from the Neotropical region (Choe 2018; Kočárek and Horká 2022, 2023a, 2023b), the knowledge of the species diversity of the Afrotropical region is likely very fragmentary.

Recently we got the chance to study samples from Cameroon and Madagascar. The single specimen from Cameroon displays characteristics which allow easy characterization and identification as a species not described yet. Characteristics of *Z.vinsoni* Paulian, 1951, to which our specimens from Madagascar belong, have been described relatively superficially by Paulian (1951). Useful diagnostic features are often found in the male postabdomen (Kočárek et al. 2020; Matsumura et al. 2020; Kočárek and Horká 2023a, 2023b), especially the genital apparatus. Therefore we described the postabdominal morphology in detail. In Zoraptera, parthenogenetic species and/or populations occur (Rafael et al. 2008). Species identification of such parthenogenetic individuals is sometimes difficult and can potentially lead to misidentification. Therefore, although the female morphology has not been well documented in taxonomic studies of the order, we also describe it in detail for *Z.vinsoni*.

## ﻿Material and methods

The holotype of *Z.komatsui* sp. nov. was fixed first in 99.5% ethanol and after extraction of DNA preserved in 70% ethanol. It was then slide-mounted in Euparal (Waldeck GmbH & Co. KG, Münster, Germany) and is deposited at Hokkaido University (Japan). We studied three apterous males, three apterous females, and one alata female of *Z.vinsoni*. One apterous female was fixed in FAE and preserved in 70% ethanol, whereas the other specimens were fixed and preserved in 70% ethanol. One female and male fixed in ethanol were macerated in 10% KOH and slide-mounted in Euparal. These slide-mounted specimens of *Z.vinsoni* are deposited at Hokkaido University, and the remaining material of the species are deposited in YM’s research collection, presently kept at Hokkaido University.

The habitus of the single specimen from Cameroon was first photographed using a Keyence VHX-7000 (Keyence Corporation, Osaka, Japan) equipped with a VH-Z20R 20–200× objective. Subsequently, all specimens used in the present study were investigated under a stereomicroscope Leica M205 C (Leica Microscopy GmbH, Wetzlar, Germany) equipped with a camera EOS 6D Mark II (Canon, Tokyo, Japan), and slide specimens were observed under a light microscope Olympus BX40 (Olympus Corporation, Tokyo, Japan) or a light microscope Zeiss Axiophot (Carl Zeiss Microscopy GmbH, Jena, Germany) equipped with a camera Olympus OM-D E-M5 Mark III. Photographs were taken at different focus planes and stacked using Zerene software (Zerene Systems LLC, WA, USA). Subsequently, line drawings were prepared using Illustrator CC and combined with photographs using Photoshop. For measurements, Fiji (Schindelin et al. 2012) was used. Confocal scanning laser microscopy (CLSM) was employed to document genitalic sclerites, and autofluorescence was visualized using a CLSM TCS SP5 (Leica Microscopy GmbH). Wavelengths of 405 nm, 488 nm, 555 nm and 639 nm were used for excitation ones, and wavelengths 420–480 nm, ≥ 490 nm, ≥ 560 nm or ≥ 640 were detected, respectively. Blue, green, red (50% saturation), and red (50% saturation) were assigned to micrographs obtained by each set of settings, and then overlaid with the maximum intensity projection using the software LAS X (Leica Microscopy GmbH).

## ﻿Results

**Family Zorotypidae Silvestri, 1913**

**Subfamily Zorotypinae Silvestri, 1913**

**Genus *Zorotypus* Silvestri, 1913**

### ﻿ Zorotypus komatsui

Taxon classificationAnimalia

Matsumura, Maruyama, Ntonifor & Beutel sp. nov.

C54F7001-185B-5D11-AAE3-F4C4918B1839

https://zoobank.org/1464A259-D721-43D8-920D-0237C612B20A

Figs 1 , 2 , 3 , 4

Zorotypus sp. 1 cameroon YK2: Matsumura et al. 2020: 352.

#### Type material.

***Holotype*.** Cameroon: apterous male, South-west region, Nyasoso, Mt Kupe, 4°50'12.5"N, 9°41'21.7"E, 16.v.2015, coll. Takashi Komatsu (depository number/ SEHU48817-48818, parts of antennae mounted on another glass slide). The male was found under a rock which is unusual for Zoraptera (see Remarks for detail).

#### Diagnosis.

Males can be easily distinguished from those of other zorapteran species from Africa by the prominently developed projections on Tx and Txi and two pairs of sensilla basiconica on Tx. The following features should be added to the diagnosis: (1) eye spots absent in apterous males; (2) antennomere ii shorter than antennomere iii; (3) posterior metafemural surface covered with seven stout spiniform bristles, two of which are distantly located on middle region and longer, and the rest arranged evenly on the proximal portion; and (4) metatibia with three stout spiniform bristles, two of them inserted apically.

#### Description.

**Apterous male.**

***Measurements*.***N* = 1. Total body length: 2.42 mm, head width 0.50 mm, head length 0.47 mm, antennal length 1.26 mm, pronotal length 0.38 mm, metafemural length 0.68 mm, metatibial length 0.79 mm, abdominal maximum width 0.49 mm, length of cerci 0.11 mm.

**Figure 1.**
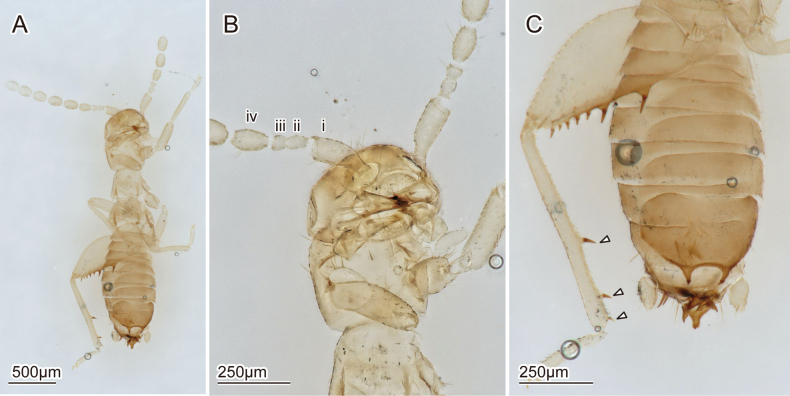
Digitalmicroscopic images of the holotype of *Zorotypuskomatsui* sp. nov. (ventral view) **A** habitus **B** head and prothorax **C** hindleg and abdomen.

***Color*.** Coloration light brown except for membranous regions and less pigmented cerci, antennae, and legs (Fig. 1). The holotype was processed with Proteinase K (Qiagen, Tokyo, Japan) and the body consequently less pigmented than in a natural condition.

**Figure 2.**
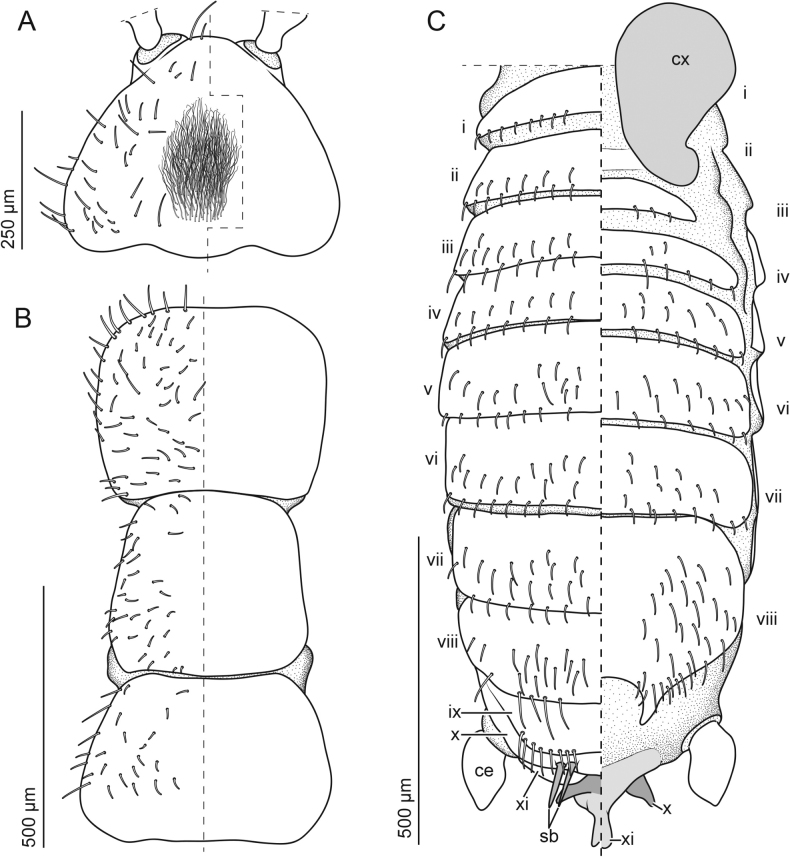
Adult *Zorotypuskomatsui* sp. nov. male **A** head, dorsal view **B** thorax, dorsal view **C** abdomen, dorsal (left) and ventral (right) views. Abbreviations: ce; cercus, cx; coxa, sb; sensilla basiconica.

***Head*** (Figs 1, 2A). Head subtriangular, without black eye spots (Figs 1, 2A); ocelli absent; cephalic chaetotaxy as in Fig. 2A; relatively long sinuate setae densely arranged on vertex, referred to as fontanelle (e.g., Delamare-Deboutteville 1951; van Ryn-Tournel 1971) (Fig. 2A). Antennae 9-segmented; antennomere i slightly curved outward, longer than wide; antennomere ii short, about 1/3 as long as antennomere i, longer than antennomere iii (Fig. 1B); antennomeres iv–ix cylindrical and longer than wide, about 2.2 times as long as antennomere ii. Labial palps three-segmented.

***Thorax*** (Figs 1, 2B). Pronotum trapezoid. Mesonotum trapezoid, as long as pronotum. Metanotum trapezoid, distinctly wider than long, shorter than mesonotum. Thoracic setation as in Fig. 2B. Legs covered with short and moderately long setae; tarsi 2-segmented and covered with moderately long setae, with small unguitractor plate apically and pair of claws; posterior and ventral profemoral surfaces with moderately long setae; protibia with moderately long setae, and bristles arranged as comb on distal two-thirds along ventral surface; anterior and ventral mesofemural surfaces with moderately long setae; mesotibia with vestiture of moderately long setae and two apical spurs; metafemur broader than profemur, wider proximally than distally; ventral metafemural surface covered with moderately long setae; posterior surfaces with seven stout spiniform bristles, two on middle region, rest evenly distributed distally and longer than central ones (Fig. 1C); metatibia with moderately long setae and three stout spiniform bristles, two of them inserted apically (Fig. 1C).

**Figure 3.**
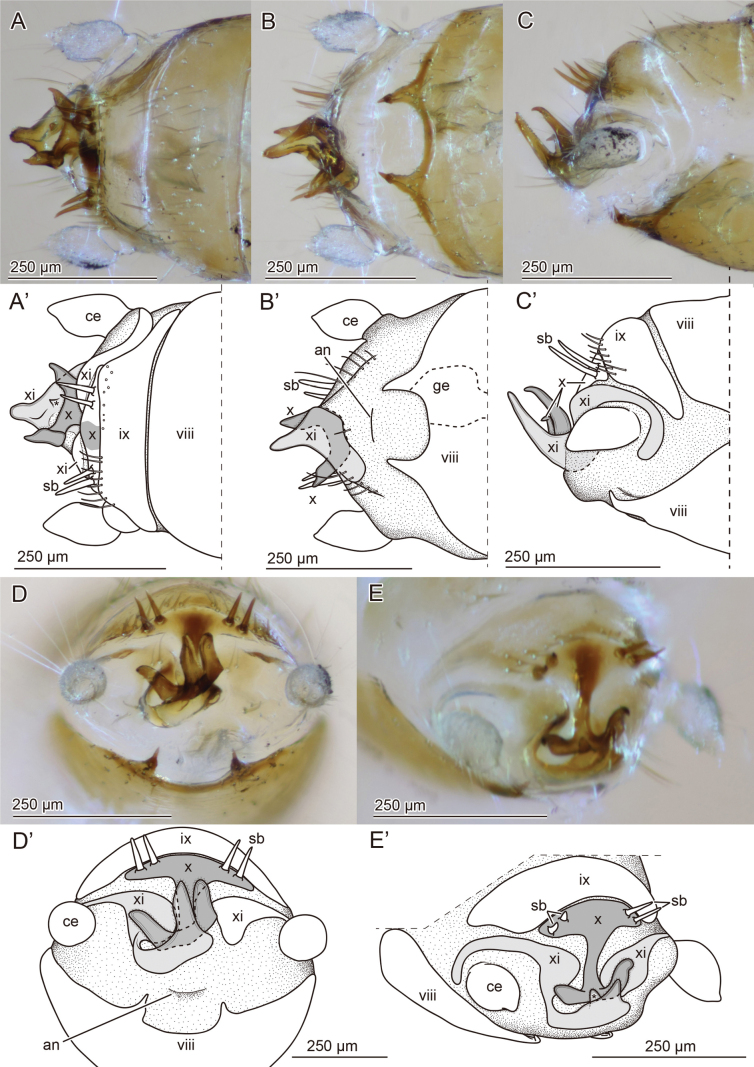
Apical region of male abdomen of *Zorotypuskomatsui* sp. nov. **A–E** micrographs **A'–E**' corresponding drawings **A–A**' dorsal view **B–B**' ventral view **C–C**' lateral view **D–D**' caudal view **E–E**' caudodorsal view. Abbreviations: an; anus, ce; cercus, ge; genitalia, sb; sensilla basiconica.

**Figure 4.**
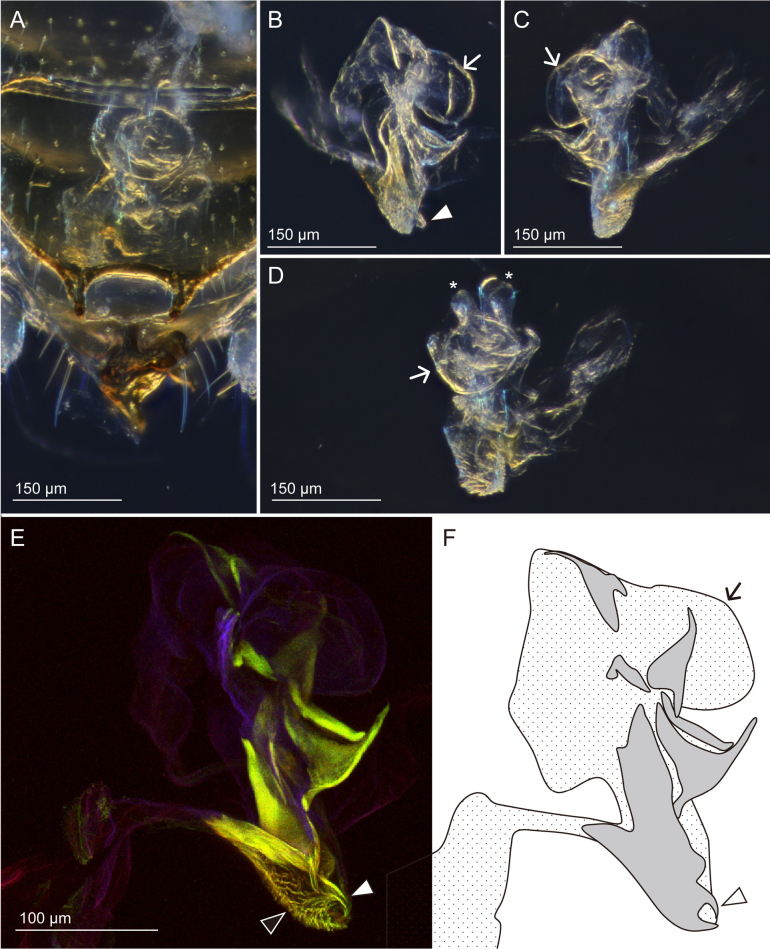
Male genitalia of *Zorotypuskomatsui* sp. nov. with six inconspicuous sclerites, one globular less sclerotized structure (arrows) and two membranous projections (asterisks) **A–D** light micrographs **E**CLSM images (white arrowhead denotes a projection, and the black one denotes microstructures) **F** schema showing positions of six sclerites **A, D** ventral view **B, C, E** lateral view **D** lateral view, the sclerites were traced on **E**.

***Abdomen*** (Figs 1, 2C, 3, 4). Chaetotaxy of abdominal tergites and sternites as in Fig. 2C. Abdominal tergum 1 (Ti) with single transverse row of short setae; Tii–vi with two transverse rows of short setae and additional setae of moderate length; Tvii with three transverse rows of moderately long setae; posterior two-thirds of Tviii covered with moderately long setae and several long setae along posterior edge; Tix trapezoidal, posteriorly narrow, with row of long setae along posterior edge; Tx with two pairs of sensilla basiconica (Fig. 2C), medially heavily sclerotized and continuing as bifurcated projection (Fig. 3); Txi partially sclerotized but partially semimembranous, with sclerotized median projection originating from left side (Fig. 3D–E'). Projections on Tx and Txi very large, superficially resembling asymmetric male genitalia; projection on Tx bifurcated, fork-like; projection on Txi triangular, with horn on dorsal side (Fig. 3E asterisk). Cerci unsegmented, conical with numerous short setae and several long and fine setae. Si scarcely sclerotized; Sii laterally weakly sclerotized; Siii with transverse row of short setae along posterior margin; Siv with two transverse rows of short setae and additionally setae of moderate length; Sv–vii with three transverse rows of short setae and setae of moderate length; Sviii wider than long, with moderately long setae evenly distributed except for anterior and middle regions; posterior margin bifurcated, tips bent dorsad (Fig. 3C, C'). Genitalia with six inconspicuous sclerites (Fig. 4, the largest sclerite with microstructures [Fig. 4, black arrowhead] and pointed protuberance [Fig. 4, white arrowheads]), one globular less sclerotized structure (Fig. 4, white arrows) and two membranous projections (Fig. 4, asterisks); highly reduced, almost vestigial (Fig. 4).

#### Distribution.

Cameroon, South-west region, Nyasoso, Mt Kupe.

#### Remarks.

The habitat of the individual we obtained was unusual for a zorapteran species. The male was found under a rock ca 30 cm long and half embedded in soil. Furthermore, the rock was located in an open relatively dry area. The collector of the specimen T. Komatsu and one of the authors (MM) tried intensively to find zorapterans in rain forests nearby, but no additional individual was found.

### ﻿ Zorotypus vinsoni

Taxon classificationAnimalia

Paulian, 1951

96C2CAD0-D7E6-55B3-81FE-6E3396D9253D

**Figure 5.**
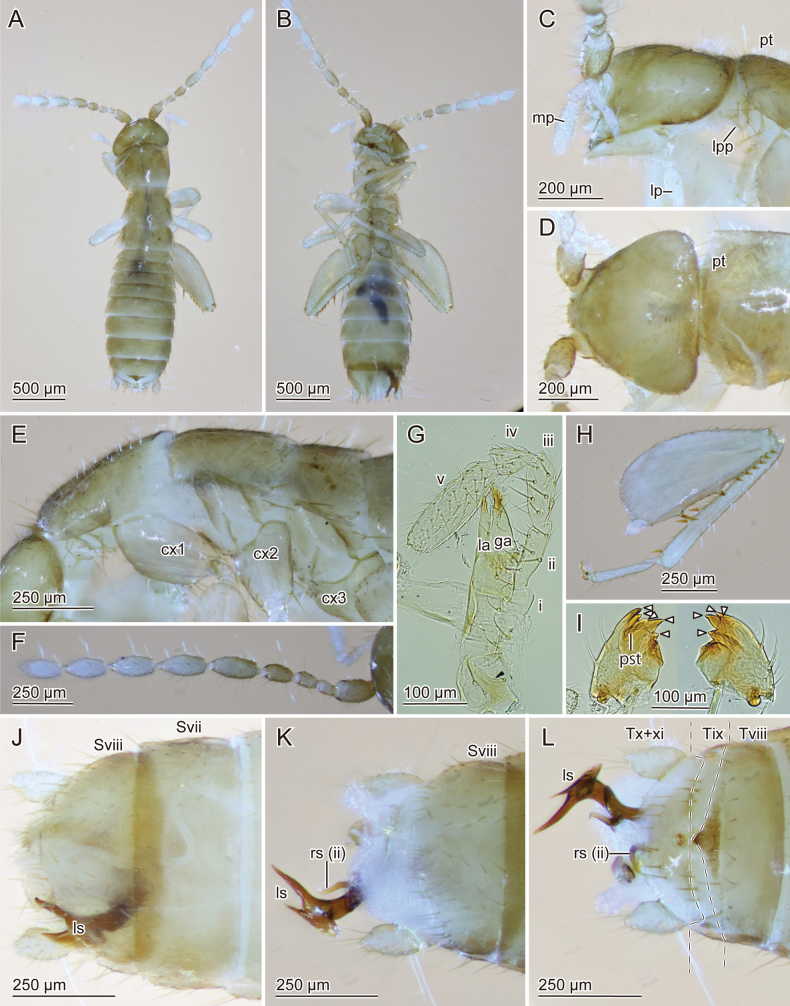
Adult *Zorotypusvinsoni* male **A, B** habitus **A** dorsal **B** ventral view **C, D** head **C** lateral **D** dorsal view **E** thorax, lateral view **F** right antenna **G** left maxilla, light microscopic image **H** right hindleg, dorsal view **I** left and right mandibles, dorsal view **J–L** postabdomen, a part of genitalia is partially extruded in **K, L** ventral view in **J, K** and dorsal view in **L**. Abbreviations: cx; coxa, ga; galea, la; lacinia, lp: labial palpus, lpp; lateral protuberance of prosternum, ls; left sclerite, mp; maxillary palpus, pst; prostheca, pt; pronotum, rs; right sclerite, S; sternite, T; tergite.

**Figure 6.**
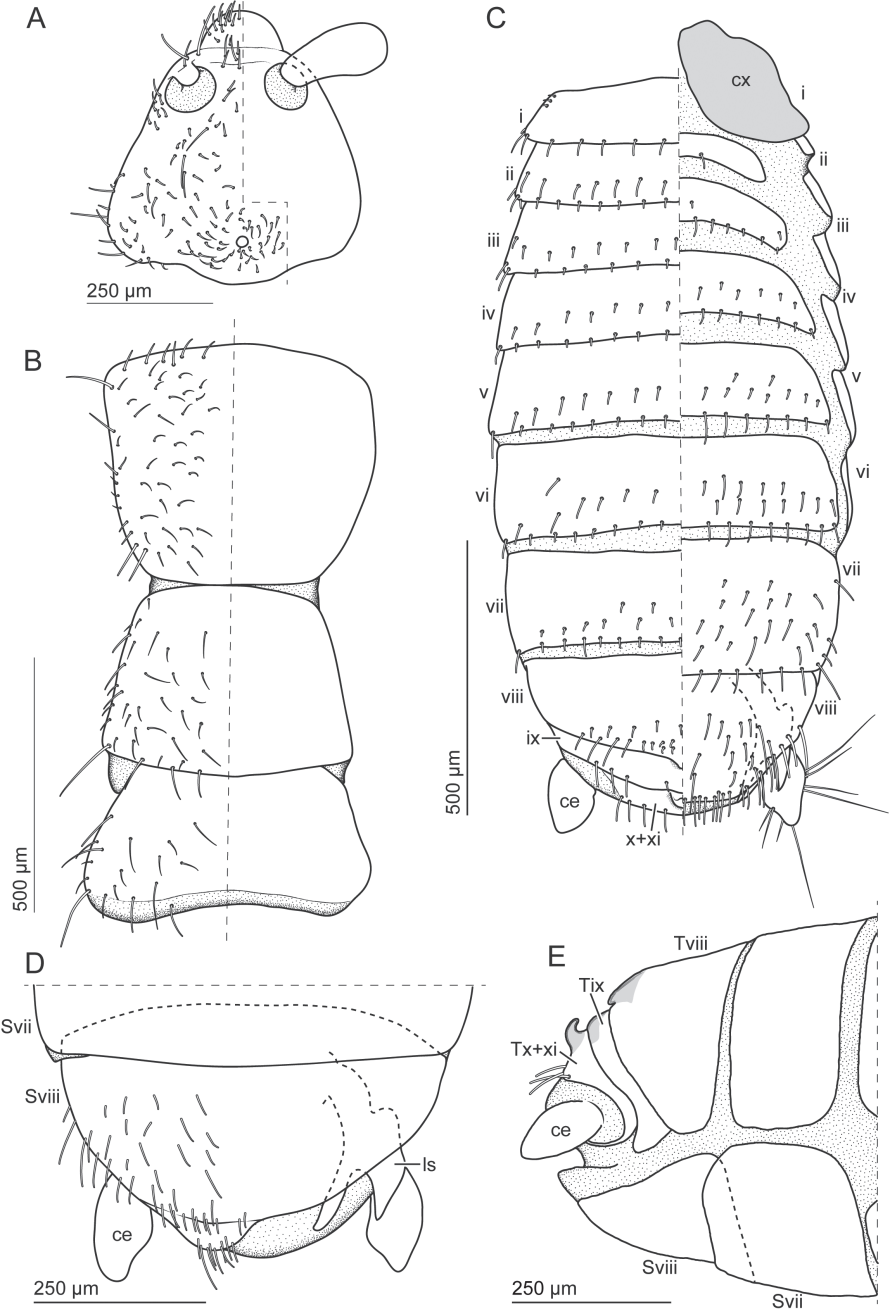
Adult *Zorotypusvinsoni* male **A** head, dorsal view **B** thorax, dorsal view **C** abdomen, dorsal (left) and ventral (right) views **D** postabdomen, ventral view **E** postabdomen, lateral view. Abbreviations: ce; cercus, cx; coxa, ls; left sclerite, S; sternite, T; tergite.

**Figure 7.**
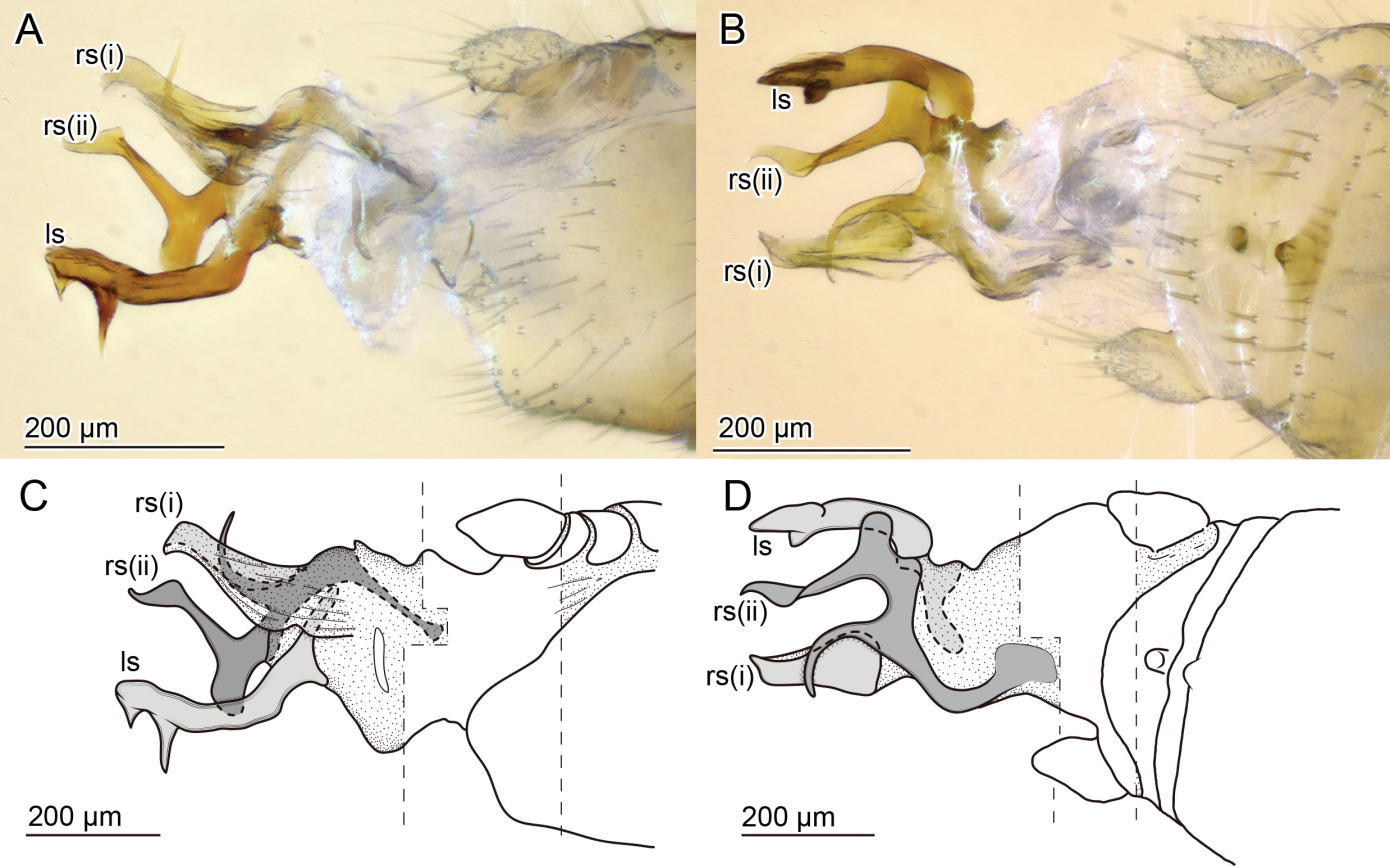
Male genitalia of *Zorotypusvinsoni***A, B** photographs **C, D** line drawings. Slightly tilt ventral view in **A, C** and dorsal view in **B, D**. Abbreviations: ls; left sclerite, rs; right sclerites, S; sternite, T; tergite.

**Figure 8.**
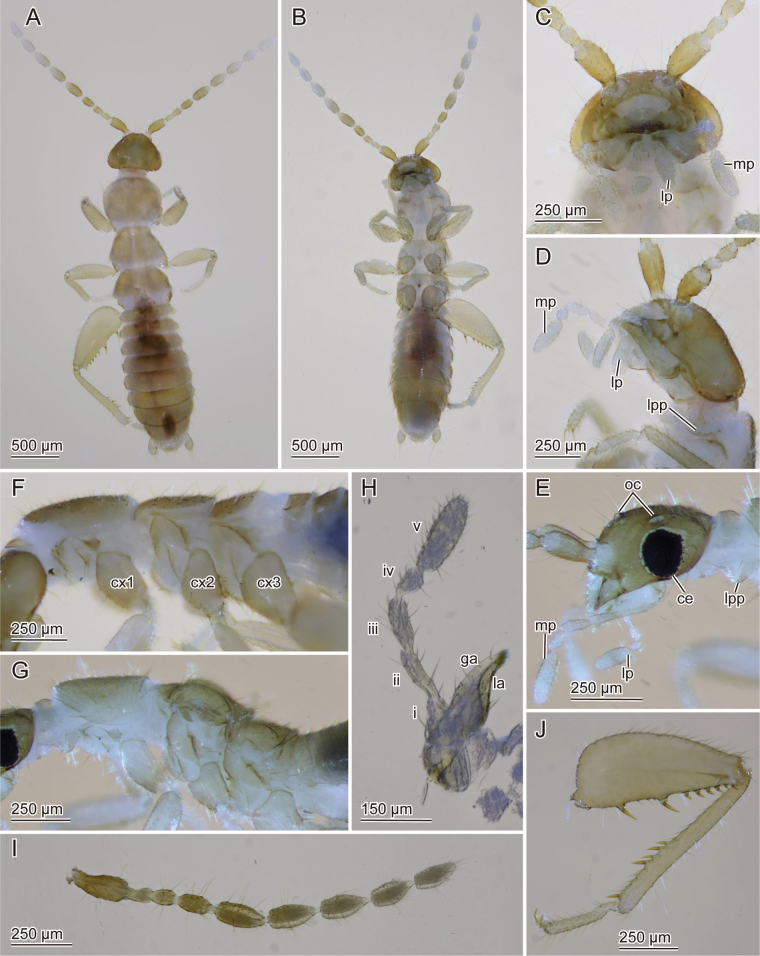
Adult *Zorotypusvinsoni* female **A, B** habitus **A** dorsal **B** ventral view **C–E** head **C** frontal **D, E** lateral view **D** wingless **E** alate **F, G** thorax, lateral view **F** wingless **G** alate **H** maxilla, light microscopic image **I** right antenna **J** right hindleg, dorsal view. Abbreviations: ce; compound eye, cx; coxa, ga; galea, la; lacinia, lp: labial palpus, lpp; lateral protuberance of prosternum, mp; maxillary palpus, oc; ocelli, pt; pronotum.

**Figure 9.**
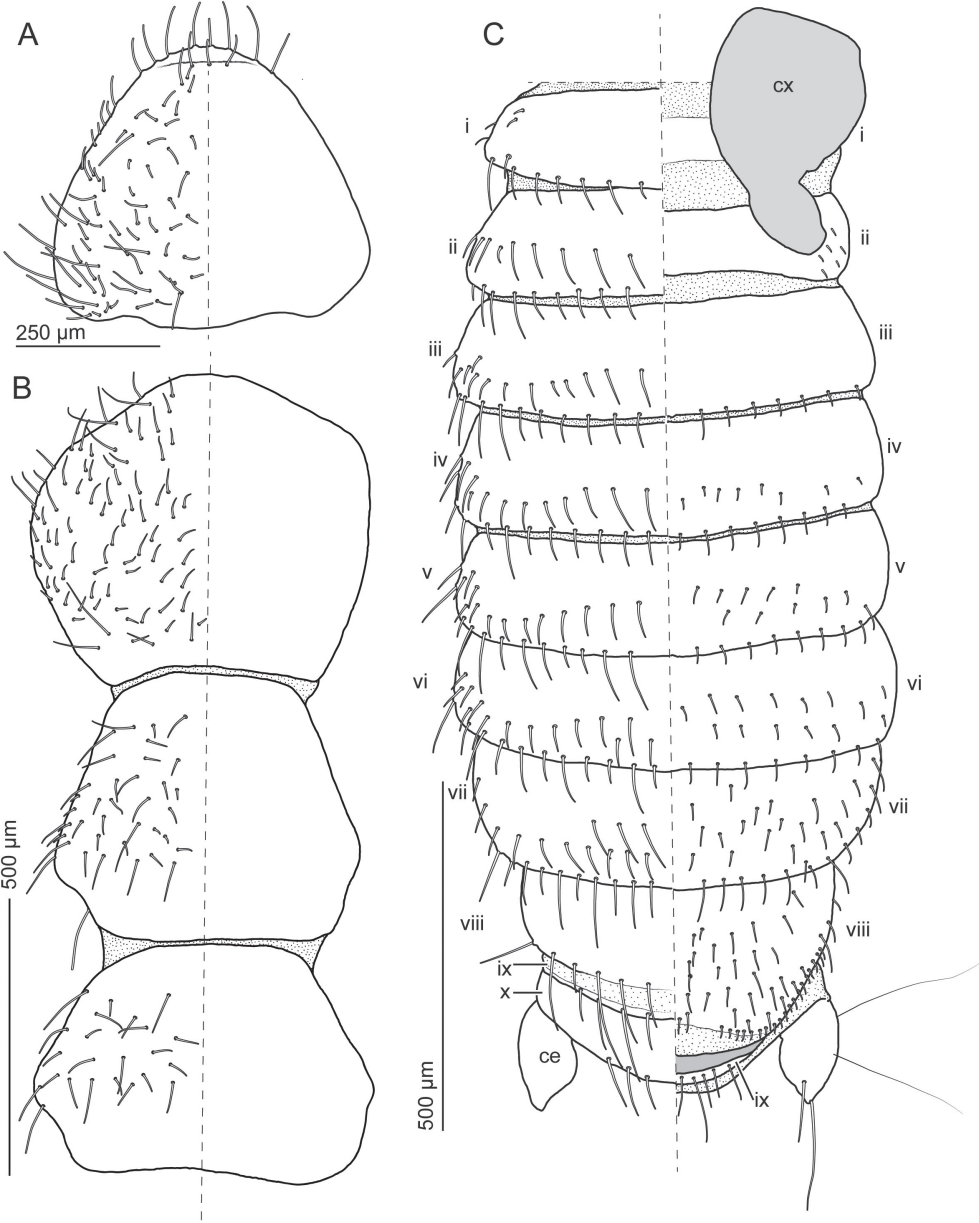
Adult *Zorotypusvinsoni* female **A** head, dorsal view **B** thorax, dorsal view **C** abdomen, dorsal (left) and ventral (right) views. Abbreviations: ce; cercus, cx; coxa.

**Figure 10.**
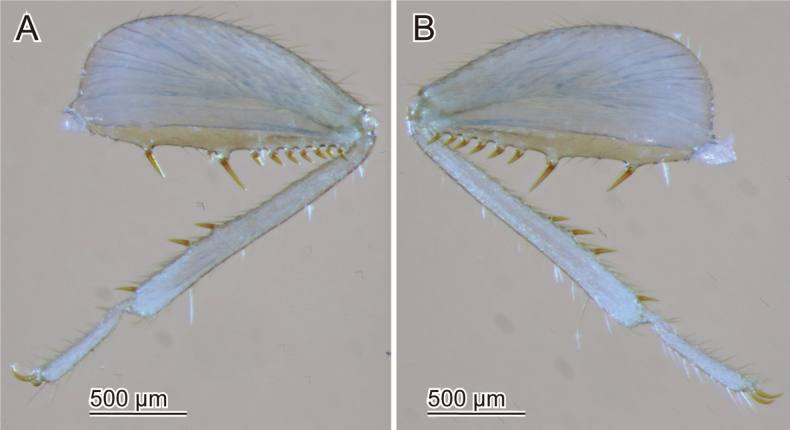
Individual variations of hindleg spurs of *Zorotypusvinsoni*. The sex of those individuals is unknown **A** right hindleg, dorsal view **B** left hindleg, dorsal view.

Figs 5 , 6 , 7 , 8 , 9 , 10

Zorotypus vinsoni : Paulian 1951: 34.
Zorotypus vinsoni : Hubbard 1990: 57.
Zorotypus vinsoni : Kočárek et al. 2020: 51.

#### Type locality.

Types were not explicitly designated by Paulian (1951), but the author described the species based on specimens collected from Maccabean forest (alt. 600 m) on Mauritius island.

#### Material examined.

Madagascar: three apterous males, three apterous females, one alata female, near Aventure trail, Andasibe NP., 18°93'60"S, 48°41'90"E, 920 m., 5.iv.2019, coll. P. Jałoszyński (depository number of slide-mounted specimens/ SEHU48819-48822).

#### Diagnosis.

According to Paulian (1951), this species can be distinguished from *Z.delamarei* only by male genitalic morphology which is characterized as follows: (1) asymmetrical; (2) left valve strongly sclerotized, abruptly bent at nearly right angle, with bifurcated apex (left branch anterolaterally expanded and shorter than right one); (3) right valve composed of two sclerites (i and ii); (4) sclerite ii less stout and bifurcated (left branch twisted apically and with basal blade-like projection, right branch curved and pointed); (5) sclerite i located close to curved and pointed branch of sclerite ii. The following features should also be added to the diagnosis: (6) eye spots absent in apterous males; (7) antennomere ii as long as antennomere iii; (8) posterior metafemural surface covered with eight to nine stout spiniform bristles, first and second long and inserted on proximal to middle region, other bristles shorter and evenly distributed on proximal area; and (9) metatibia with three to four stout spiniform bristles, two of them inserted in middle region.

#### Description.

**Apterous male.**

***Measurements*.***N* = 2. Total body length: 2.58–3.25 mm, head width 0.53–0.54 mm, head length 0.43–0.46 mm, antennal length 1.36–1.53 mm, pronotal length 0.45–0.47 mm, metafemoral length 0.75 mm, metatibial length 0.77 mm, abdominal maximum width 0.62–0.66 mm, length of cerci 0.13 mm.

***Color*.** Coloration light brown except for whitish membranous regions and less pigmented cerci, antennae, and legs (Fig. 5).

***Head*** (Figs 5, 6A). Head subtriangular, slightly wider than pronotum, without black eye spots (Fig. 5D); ocelli absent; cephalic chaetotaxy as in Fig. 6A; short setae arranged in dense, oval group on vertex, referred to as fontanelle (e.g. Delamare-Deboutteville 1951; van Ryn-Tournel 1971) (Figs 5D, 6A), with pore between them (possibly gland opening). Antennae 9-segmented, with distal three or four antennomeres less pigmented than others (Fig. 5F); antennomere i slightly curved outward, longer than wide; antennomere ii short, about half as long as antennomere i, equal to antennomere iii; antennomere iv slightly longer than antennomeres ii and iii, antennomeres v–ix longer than wide (Fig. 5F). Left mandible with five apical teeth and well-developed molar region and prostheca on ventral surface (black arrow in Fig. 5I); right mandible with four apical teeth and well-developed molar region but without prostheca. Maxillae with distinctly separated lacinia and galea, both with densely arranged setae on distal part; maxillary palps five-segmented (Fig. 5G). Labial palps three-segmented (Fig. 5C).

***Thorax*** (Figs 5, 6B). Pronotum trapezoid, slightly narrowed posteriorly; pro­sternum with peg-like anterolateral protuberances (Fig. 5C). Mesonotum trapezoid, slightly shorter than pronotum. Metanotum trapezoid, distinctly wider than long, shorter than mesonotum. Thoracic setation as in Fig. 6B. Legs covered with short and moderately long setae; tarsi 2-segmented with small unguitractor plate and hooked claws; ventral and posterior profemoral surfaces covered with short setae; protibia with short setae and bristles arranged as antenna cleaning organ on proximal two-thirds along ventral surface; mesotibia with vestiture of moderately long setae and two apical spurs; metafemur broader than pro- and mesofemora, wider proximally than distally; ventral surface evenly covered with moderately long setae; posterior surface with eight to nine stout spiniform bristles (Fig. 5H), first and second long and inserted on proximal to middle region, other bristles shorter and evenly distributed on distal area; metatibia with moderately long setae and three to four stout spiniform bristles.

***Abdomen*** (Figs 5, 6C, 7). Abdominal tergite 1 (Ti) with single transverse row of short setae, and few small setae laterally (Fig. 6C); Tii–vii with two transverse rows of short setae and additionally setae of moderate length, and pair of long erect setae inserted at posterior corner; Tviii medially sclerotized, with posterior projection and two transverse rows of short setae and setae of moderate length (Fig. 6C, E); Tix short and medially sclerotized, with transverse row of short setae and setae of moderate length; Tx + xi not visible externally, medially sclerotized, with anterior curved projection; with transverse row of setae of moderate length and with lateral ends enclosing cerci (Fig. 6C, E). Cerci unsegmented, conical, with one subapical long seta, few relatively long subapical setae, and numerous short and fine setae. Si scarcely sclerotized; Sii evenly sclerotized and with pair of setae; Siii with transverse row of short setae along posterior margin; Siv–v with two transverse rows of short setae and setae of moderate length; Svi with three transverse rows of short setae and setae of moderate length; posterior two-thirds of Svii with evenly distributed short and moderately long setae; Sviii semicircular with slightly asymmetric posterior margin, often folded and invisible (Fig. 6D), posterior margin with densely arranged longer setae. Genitalia asymmetrical (Figs 5J–L, 7); left valve strongly sclerotized, abruptly bent at nearly right angle, with bifurcated apex (left branch anterolaterally expanded and shorter than right one); right valve composed of two sclerites (i and ii in Fig. 7), sclerite ii less stout and bifurcated (left branch twisted apically and with basal blade-like projection, right branch curved and pointed); sclerite i located close to curved and pointed branch of sclerite ii.

**Apterous female** (Figs 8, 9).

***Measurements*.***N* = 2. Total body length: 2.82–3.00 mm, head width 0.57–0.60 mm, head length 0.38–0.55, antenna length 1.62–1.65 mm, pronotal length 0.44–0.51 mm, metafemoral length 0.76–0.80 mm, metatibial length 0.70–0.76, abdominal maximum width 0.67–0.79 mm, length of cerci 0.12–0.15 mm.

***Morphology*.** Similar to apterous male. Oval group of setae on vertex with pore between them absent (Figs 8A, 9A). Setae on abdominal tergites generally longer and lateral tergal regions setose; Tviii uniformly sclerotized, with transverse row of long setae (Fig. 9C); Tix only posteriorly sclerotized, with setae of moderate length (Fig. 9C); Tx uniformly sclerotized (Fig. 9C). Si only slightly sclerotized; Sii with short setae laterally; posterior margin of Sviii membranous, with more or less evenly distributed moderately short setae.

**Alata female** (Fig. 8E, G).

***Morphology*.** Similar to apterous female. Darker brown in coloration. Compound eyes and three black ocelli present. Scuto-scutellar suture distinctly visible on mesonotum and metanotum (Fig. 8G).

#### Distribution.

Andasibe on Madagascar (newly found record here), Maccabean forest (alt. 600 m) in Mauritius island. Under bark.

#### Variation

**(Figs 5H, 7J, 10).** In two cases with one or two additional slender spiniform bristles on metatibia.

#### Validity of the species identification.

Until we obtained specimens of *Z.vinsoni* from Madagascar, the presence of *Z.vinsoni* was reported only from Mauritius island (Paulian 1951). In contrast, *Z.delamarei* Paulian, 1949 was known from Madagascar (Paulian 1949; Kočárek et al. 2020). According to Paulian (1951), *Z.delamarei* and *Z.vinsoni* are only distinguishable by male genitalic morphology. Our observations clearly suggest that the genitalic morphology of our study samples from Madagascar matches the documentation of the male genitalia in *Z.vinsoni* in Paulian (1951, fig. 3). Therefore, we consider that our identification should be justified, although we have not been able to get access to original research materials used by Paulian (1949, 1951).

The collection site of our study samples of *Z.vinsoni* is about 85 km west of the third locality of specimens of *Z.delamarei* used in Paulian (1949). The three locations in the northern half of Madagascar where his samples were collected are listed below:

1. 10 km S.E. Ambanja, in bast fibres, on the road of Haut-Sambirano.
2. Forest of Ambohitantely, 140 km. N.NW. of Tananarive, in small, slightly degraded grove of trees.
3. Forest of la Mandraka, 80 km. W. of Tananarive, in degraded forest.

The samples of *Z.delamareii* used in Kočárek et al. (2020) were collected nearby (less than 50 km from our sample collection site) from the following localities (P. Kočárek, pers. comm.):

1. Central Madagascar Andasibe-Mantadia NP., Mantadia, circuit „Eulophia“, 22–23.i.2017, 18°48'16"S, 48°25'43"E, 958 m, coll. P. Baňař (collected with an interceptor trap).
2. Central Madagascar Andasibe-Mantadia NP., Mantadia, end of circuit „Eulophia“, 22.i.2017, 18°79'87"S, 48°42'78"E, 970 m, coll. P. Janšta (collected from dead trunk over a stream).
3. Central Madagascar Andasibe-Mantadia NP., Mantadia circuit „Eulophia“, 22.i.2017, 970 m, 18°80'37"S–18°79'87"S, 48°42'92"E–48°42'78"E, coll. P. Janšta.

Considering the hitherto known localities, these two closely related species could occur either parapatrically or sympatrically in Madagascar.

## ﻿Discussion

In the present study, we described the seventh species of Zoraptera from Africa, *Z.komatsui*. We also report a new distribution record on Madagascar for *Z.vinsoni*, which was previously collected on Mauritius island. The discoveries were made during short excursions in Cameroon and Madagascar, both not focused on collecting zorapterans. This clearly suggests that the present knowledge of the species diversity of the Afrotropical region is very fragmentary. Consequently, a thorough exploration of the African zorapteran fauna should have a high priority.

The new distribution record of *Z.vinsoni* tentatively suggests that the expansion of the distribution of this species occurred via dispersal from Madagascar to Mauritius island. Mauritius island arose 8.9 Ma by a volcanic eruption, and a more recent series of volcanism began 1 Ma (Moore et al. 2011). Therefore, it is conceivable that *Z.vinsoni* and *Z.delamarei* were split from a common ancestor in Madagascar, and that the population on Mauritius island was established by a relatively small number of individuals from Madagascar. Both alate and wingless morphs occur in Zoraptera, and the thoracic musculature including flight muscles is well developed in the former (Friedrich and Beutel 2008). However, it is highly unlikely that zorapterans covered the distance from Madagascar to Mauritius island by active flight. Passive dispersal by strong winds may have played a role or also transport via driftwood. This is in contrast to the typical pattern in Zoraptera, i.e., the establishment of distribution patterns by vicariance (Matsumura et al. 2020).

The phylogenetic positions of our study species belonging to the genus *Zorotypus* s. str. (Kočárek et al. 2020) can be only partially inferred. In a previous molecular phylogenetic study, *Z.komatsui* was included as “*Zorotypus* sp. 1 cameroon YK2” and was placed as sister to a clade comprising *Zorotypusasymmetristernum* Mashimo, 2019 from Kenya + *Z.shannoni* Gurney, 1938 from Brazil (Matsumura et al. 2020). A possibly close relative of *Z.vinsoni*, *Z.delamarei*, was included, but no other *Zorotypus* species were included in the molecular phylogenetic study of Kočárek et al. (2020). Therefore, the phylogenetic relationships of the species treated in the present contribution remain ambiguous. One of the most variable characters among species in Zoraptera is the male genital apparatus, and it is apparent that this character system carries phylogenetic signal (Kočárek et al. 2020; Matsumura et al. 2020). However, the homology of genitalic sclerites among zorapterans has not been established yet. The terminology applied in previous studies is not stable. Authors of the present study (YM and RGB) have been dealing with this problem, but a clarification of this exceptionally complicated issue is clearly beyond the scope of the present study.

Although we could only study a single male specimen from Cameroon, this individual displays characteristics which allow for easy distinction from other species. Pronouncedly developed projections on Tx and Txi, the presence of large sensilla basiconica on abdominal tergite 10 (Tx), and vestigial genitalia are a noteworthy and unique combination of features of this newly described species. The male genitalia are scarcely recognizable without dissection (in this case the holotype), whereas a part of the male genitalia of *Z.vinsoni* is visible through sternite 8. A similar combination of features, except for the presence of sensilla basiconica on Tx, was reported for *Spermozorosimpolitus* (Mashimo et al., 2013) from Malaysia (Mashimo et al. 2013). In all zorapteran species whose mating posture was observed, males are almost always supine and hanging on the tip of the female abdomen (Shetlar 1978; Choe 1992, 1995, 1997, 2018; Mashimo et al. 2011; Dallai et al. 2013; Wang et al. 2016). The exception is *S.impolitus*, where secondary external sperm transfer takes place (Dallai et al. 2013). The similar combination of rudimentary male genitalia and well-developed postabdominal projections in *S.impolitus* and *Z.komatsui* tentatively suggests that the newly described species also performs external sperm transfer. If this is the case, this highly unusual reproductive behavior must have evolved at least two times independently within Zoraptera, since *S.impolitus* belongs to the subfamily Spermozorinae, sister to Zorotypinae to which *Z.komatsui* belongs (Kočárek et al. 2020; Matsumura et al. 2020). This issue is speculative with the presently available information, but certainly of great interest when mating has been observed and documented.

Considering that the male genitalia of *S.impolitus* are not used for insertion in the female genital tract, it appears likely that the prominent postabdominal projections are involved in the early process of mating, for instance, coercive opening of the female genital orifice. In *Spiralizoroscaudelli* (Karny, 1927), the only species whose genital coupling was described in detail, a small projection comes into contact with the female postabdomen (Matsumura et al. 2014). However, its function remains unknown. In *Z.komatsui*, the projection of Tx is fork-like and the additional projection of Txi carries a horn-like structure on the dorsal side. It is conceivable that both combined function as a grasping organ. The function of the extremely large postabdominal projections of *Z.komatsui* needs to be investigated further.

In the other species we studied here, *Z.vinsoni*, we could compare the female and male. We found variation in a frequently used diagnostic character, i.e., the number of spiniform bristles on the hindleg. When male and female specimens are obtained, only males are usually documented in detail including illustrations. Our figures show that the body setation can also differ between the sexes. Since Zoraptera has retained mainly plesiomorphic features with respect to the groundplan of Neoptera (Friedrich and Beutel 2008; Beutel et al. 2014), our detailed documentation should be helpful to identify cryptic species, as demonstrated in recent studies (Kočárek and Horká 2023a, 2023b).

## Supplementary Material

XML Treatment for Zorotypus komatsui

XML Treatment for Zorotypus vinsoni

## ﻿Additional information

### Author ORCIDs

Yoko Matsumura https://orcid.org/0000-0002-3438-2161

Munetoshi Maruyama https://orcid.org/0000-0003-4531-1008

Rolf G. Beutel https://orcid.org/0000-0002-0433-7626

### Data availability

All of the data that support the findings of this study are available in the main text.
